# The Influence of Host miRNA Binding to RNA Within RNA Viruses on Virus Multiplication

**DOI:** 10.3389/fcimb.2022.802149

**Published:** 2022-04-21

**Authors:** Lin Lei, Anchun Cheng, Mingshu Wang, Renyong Jia

**Affiliations:** ^1^ Research Center of Avian Disease, College of Veterinary Medicine, Sichuan Agricultural University, Chengdu, China; ^2^ Institute of Preventive Veterinary Medicine, Sichuan Agricultural University, Chengdu, China; ^3^ Key Laboratory of Animal Disease and Human Health of Sichuan Province, Sichuan Agricultural University, Chengdu, China

**Keywords:** miRNA, RNA virus, RISC complex, argonaute2, flavivirus

## Abstract

microRNAs (miRNAs), non-coding RNAs about 22 nt long, regulate the post-transcription expression of genes to influence many cellular processes. The expression of host miRNAs is affected by virus invasion, which also affects virus replication. Increasing evidence has demonstrated that miRNA influences RNA virus multiplication by binding directly to the RNA virus genome. Here, the knowledge relating to miRNAs’ relationships between host miRNAs and RNA viruses are discussed.

## Introduction

The discovery of microRNA (miRNA) began with *Caenorhabditis elegans* in the 1990s, in which lin-4, a small non-coding RNA (ncRNA), was discovered (Wightman et al., 1993; Moss et al., 1997). Later, scientists found that lin-14 and let-7 from *C. elegans* were able to bind to the 3' untranslated region (3’UTR) of post-transcriptional products, thus regulating gene expression in order to control the development of *C. elegans* (Hong et al., 2000; Reinhart et al., 2000). Since then, scientists have located miRNA in a variety of organisms, ranging from plants to animals, and most of the miRNAs were evolutionarily conserved. After miRbase (http://www.mirbase.org/) had been established, it became possible to search the database for miRNA sequences from different species.

In eukaryotic organisms, miRNAs regulate the post-transcriptional expression of host and virus genes by degrading mRNA and inhibiting protein translation. Some scholars suggest that this mechanism may have originally evolved from a small RNA-mediated antiviral defense system that now coordinates various developmental processes and is strongly linked to cellular homeostasis (Aguado and TenOever, 2018). Because of this, miRNA expression is also susceptible to disease occurrence *via* virus invasion and other mechanisms. Furthermore, the expression of miRNAs in the organism changes following viral infection, suggesting that they may be involved in the virus infection process itself or in the innate immunity of the organism as modulators of cellular gene post-transcriptional expression (Zhang et al., 2016; Pu et al., 2019; Diallo et al., 2021). In addition to regulating the expression of intracellular host genes, miRNA can also influence the replication and pathogenesis of RNA viruses by binding directly to the RNA virus genome (Chen et al., 2011; Trobaugh et al., 2014; Trobaugh and Klimstra, 2017). Moreover, some special host miRNAs bind to RNA within RNA viruses and affect the spatial structure of this RNA to influence viral replication (Li et al., 2013). The role of miRNAs on RNA virus genomes has the potential to treat viruses; therefore, the roles of miRNAs on RNA viral genomes are both summarized and discussed.

## Interactions Between miRNAs and Viruses

In plants, worms, and insects, RNA interference (RNAi) is an ancient, dominating, and strong antiviral defense mechanism, whereas RNAi plays an antiviral role only in undifferentiated embryonic stem cells in vertebrates (Maillard et al., 2019). The antiviral effects in vertebrates depends heavily on interferon (IFN)-mediated antiviral responses, such as type I IFN and the IFN-stimulated genes (ISGs) (Mazewski et al., 2020). Even so, the vertebrate RNAi system is still vital for the body, mainly relying on tissue- and cell-specific miRNAs to bind mRNA, thus inhibiting its translation or reducing its stability relating to the regulation of cellular protein expression during the growth, development, reproduction, and death of organisms (Maillard et al., 2013). Furthermore, the expression of miRNAs is susceptible to transcriptional efficiency, epigenetic, intracellular homeostasis, and extracellular environmental influences, leading to time-specific and tissue-specific miRNA expression (Catalanotto et al., 2016). There is evidence that the invasion of microorganisms, such as bacteria and viruses, can lead to the variable miRNA expression profiles observed in cells (Trobaugh and Klimstra, 2017).

As for the virus, we know that many experimental results have shown that a virus infection can alter the miRNA expression profiles of host cells (Skalsky and Cullen, 2010; Fu et al., 2019; Seong et al., 2020). Due to the fact that differing changes with an intracellular miRNA expression profile are caused by different viruses, we can use the specific expression of some specific miRNAs in cells as specific biomarkers of viral infection (Atherton et al., 2019; Biswas et al., 2019). Understanding these changes following virus pathogenesis helps us to learn more about the characteristics of virus infection in different hosts or tissues and to seek meaningful miRNAs within the changing miRNA expression profiles (Ninomiya et al., 2016). These changes in miRNA expression profiles could be a strategy used by RNA viruses when escaping from the host cell immune system through the fine-tuning of miRNA on intracellular protein expression, or it could be an immune reaction, meaning that the body perceives the invasion of RNA viruses and fine-tunes immune-related genes through the miRNA system to coordinate the whole immune response. Since miRNA can bind to mRNA and regulate its translation, it is reasonable to suggest that the positive RNA from RNA viruses, which carry translatable genetic information, could be targeted and regulated by the host miRNAs (Zheng et al., 2018; Li et al., 2019; Chen et al., 2021). Up until now, there have been numerous experiments conducted to try and confirm this hypothesis. In addition, studies have found that some viruses actually produce miRNAs during an infection in order to manipulate host genes or RNA genes, thus helping self-replication, mostly noted in DNA viruses. For example, as a herpesvirus, Epstein-Barr Virus (EBV) was found to encode miRNA (Cai et al., 2006; Amoroso et al., 2011). Most herpesviruses are believed to encode miRNAs based on their genome size and the number of proteins they encode (Amoroso et al., 2011; Grundhoff and Sullivan, 2011). To date, the role of miRNAs encoded by RNA viruses, as opposed to those encoded by DNA viruses with distinct characteristics, remains controversial. So far, miRNAs have been identified in several RNA virus families, including Retroviridae, Orthomyxoviridae, Flaviviridae, Filoviridae, and Coronaviridae (Grundhoff and Sullivan, 2011; Nanbo et al., 2021). In summary, both the virus and the host can regulate the viral or host genes and thus control the virus proliferation process by encoding miRNAs.

## The Process of miRNA Biogenesis

**Figure 1:**
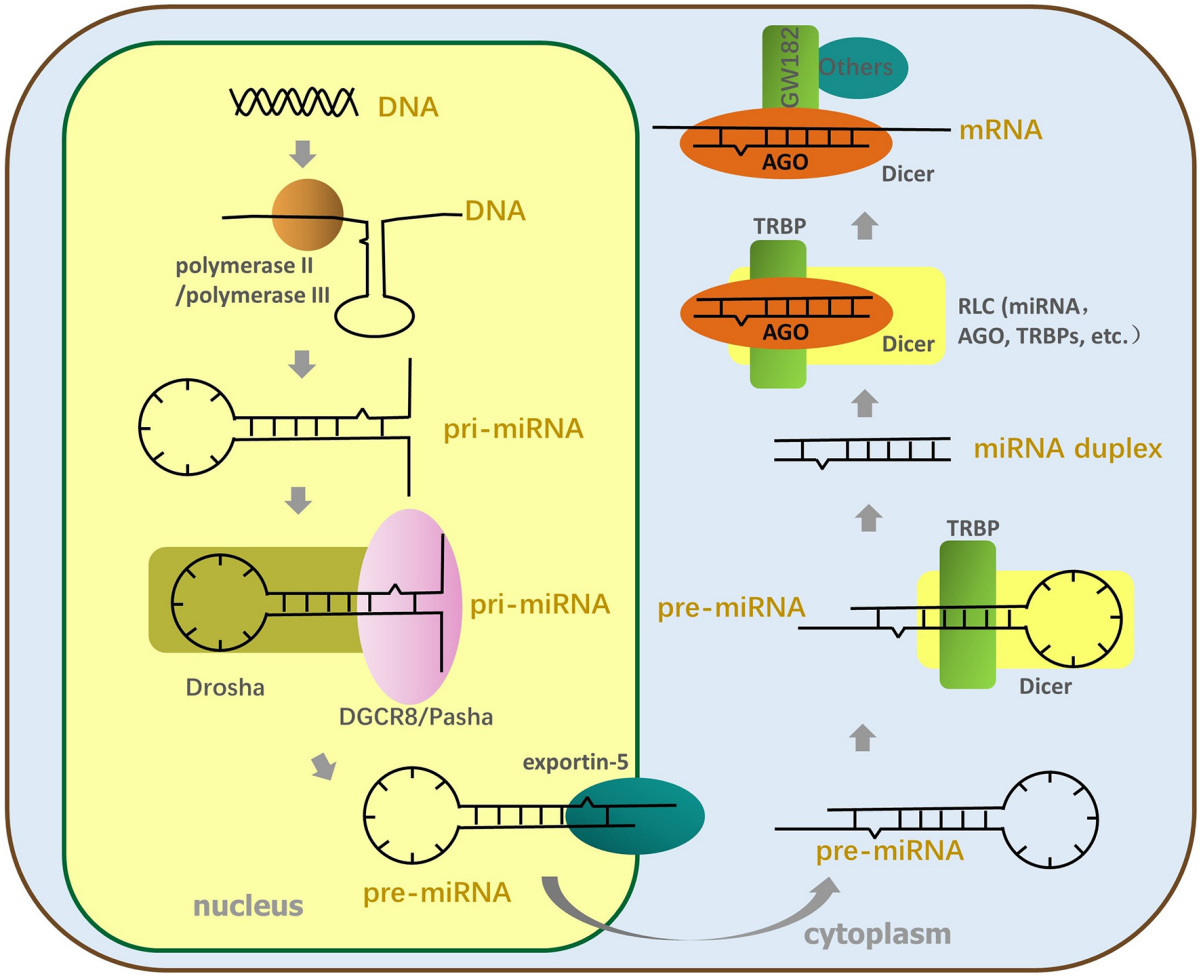
The process of miRNA formation. The miRNA genes are transcribed for pri-miRNA by polymerase II or polymerase III; then, Drosha and DGCR8 split the pri-miRNA to form SL pre-miRNA, transferred to the cytoplasm by the export receptor, exportin-5. Next, the TL element of pre-miRNA is cut off by Dicer and TRBP to produce miRNA duplex. The miRNA duplex is transferred to RLC constituted by AGO, Dicer, TRBP, and so on and is then unfastened twice. The end, mature single-stranded miRNA enters RISC, and AGO of RISC recruits downstream factors to perform RNA interference.

As shown in **Figure 1**, the miRNA genes are transcribed for primary miRNA (pri-miRNA). This occurs mainly *via* polymerase II and partly by polymerase III, which includes thousands of nucleotides and contains at least a hairpin structure (Ha and Kim, 2014). MiRNAs can be transcribed for the intron clusters of pre-mRNA, independent gene units, or long noncoding RNA (Stavast and Erkeland, 2019). Next, a “microprocessor” complex, mainly composed of the RNase III enzyme Drosha and DiGeorge critical region 8 (DGCR8) (named DGCR8 in mammals and Pasha in other animals), cuts the pri-miRNA to form stem-loop (S-L) precursor miRNAs (pre-miRNA) 60–90 nt long in the nucleus (Lee et al., 2003); then, the pre-miRNA is transferred into the cytoplasm using the export receptor, exportin-5 (Ohrt et al., 2006). The complex composed of Dicer (which belongs to the RNase III family) and transactivation-responsive RNA-binding protein (TRBP) cleaves the pre-miRNA apical loop within the cytoplasm to form double-stranded miRNAs about 22 nt in length (Bennasser et al., 2011; Kim et al., 2016). After the miRNA duplex has been transferred to the RNA-induced silencing complex, loading complex (RLC), which is mainly constituted by Argonaute (AGO) protein, and other proteins (involving translin-associated factor X, TRANSLIN and heat shock protein 90 in addition to others), the RNA duplex structure is unwound, and the passenger strand is ejected (Ye et al., 2011; Sahu et al., 2014). Different AGO proteins have distinct functions and cleavage activities that are probably determined by the PIWI domain of the AGO protein (Tang, 2005), and based on the cleavage activity of AGO, RNA-induced silencing complex (RISC) is divided into non-cleaving RISC and cleaving RISC. Subsequently, the miRNA-RISC (miRISC) complex recruits downstream factors, such as glycine–tryptophan protein of 182 kDa (GW182), including the trinucleotide repeat-containing gene 6A–6C (TNRC6A–TNRC6C), to mediate gene silencing associated with RNAi (Ding and Han, 2007; Fabian and Sonenberg, 2012).

## miRNAs Regulate Gene Expression

**Figure 2:**
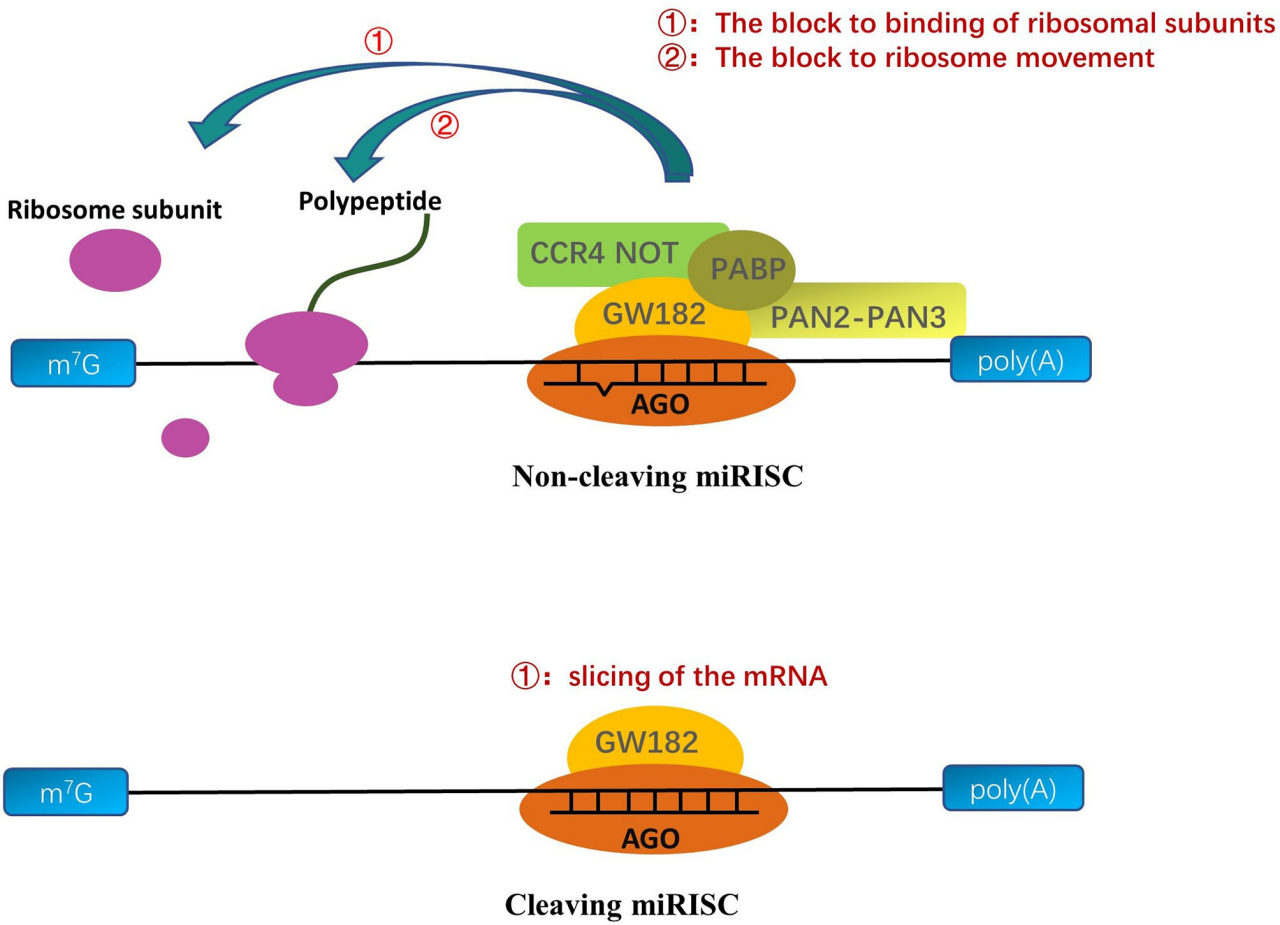
A molecular understanding of miRNA-mediated gene silencing. When the miRNA sequences are partly complementary to the targeted RNA sequences, the AGO protein binds to GW182; then, the complex recruits PABP, CCR4 NOT, and PAN2-PAN3 to interferences’ ribosome movement on the mRNA/viral RNA (vRNA) or prevents the binding of ribosomal large and small subunits to inhibit translation. When the miRNA sequences are perfect complementary to the targeted RNA sequences, cleaving-miRISC composed of miRNA and AGO protein cuts and degrades the targeted transfer RNA to inhibit translation.

In general, the seed sequence (the 2-7 nucleotide sequences of the 5’ end of mature single-stranded miRNA) helps the RISC complex target transcripts through the principle of base complementation (Chendrimada et al., 2007; Eichhorn et al., 2014). But, in fact, the other bases on some miRNAs can also help the RISC complex to locate mRNA (Lim et al., 2005; Zheng et al., 2013; Agarwal et al., 2015; Zhang Y. et al., 2017; Jiang et al., 2020). After the formation of miRNA into miRISC complexes, the methodologies of the miRNAs mainly depend on the AGO proteins of the miRISC and the degree of complementarity between the targeted mRNA sequence and the core sequence of miRNA (**Figure 2**) (Tang, 2005; Loeb et al., 2012). When the transcript target and core sequence of the miRNA are perfectly complementary for cleaving miRISC, miRISC performs the slicing of the mRNA and efficiently degrades the mRNA (Tang, 2005; Ipsaro and Joshua-Tor, 2015). But, in animals, the transcript targets rarely provide complete complementary sequences to the core sequence of the miRNA, and AGO proteins do not all have cleavage activity, which precludes mRNA cleavage by AGO proteins (Ipsaro and Joshua-Tor, 2015). Owing to the fact that AGO proteins are insufficient alone to mediate silencing, miRISC uses the GW182 protein as a platform to recruit other proteins (Takimoto et al., 2009; Kobayashi and Tomari, 2016; Sheu-Gruttadauria and MacRae, 2018). The GW182 proteins have two special structures: an amino-terminal AGO-binding domain (ABD), which binds to AGO, and a silencing domain (SD), which interacts with silencing effectors such as cytoplasmic poly(A)-binding protein (PABP), the cytoplasmic deadenylase complexes PAN2-PAN3, and CCR4 NOT (Guo et al., 2010; Braun et al., 2013). Some scholars hold the view that the interaction of these proteins with GW182 proteins interferes with the PABPC1-eIF4G interaction, which results in a decline in translational efficiency and distorts the conformation of transfer RNAs, making the 5’ cap and poly(A) tail of mRNA more readily available to utilize to degrade mRNA (Elkayam et al., 2017; Niaz and Hussain, 2018). In contrast, there is another view that the interactions between PABPC and GW182 catalyze the miRNA-mediated deadenylation of target transcripts (Meijer et al., 2013; Niaz and Hussain, 2018).

**Figure 3:**
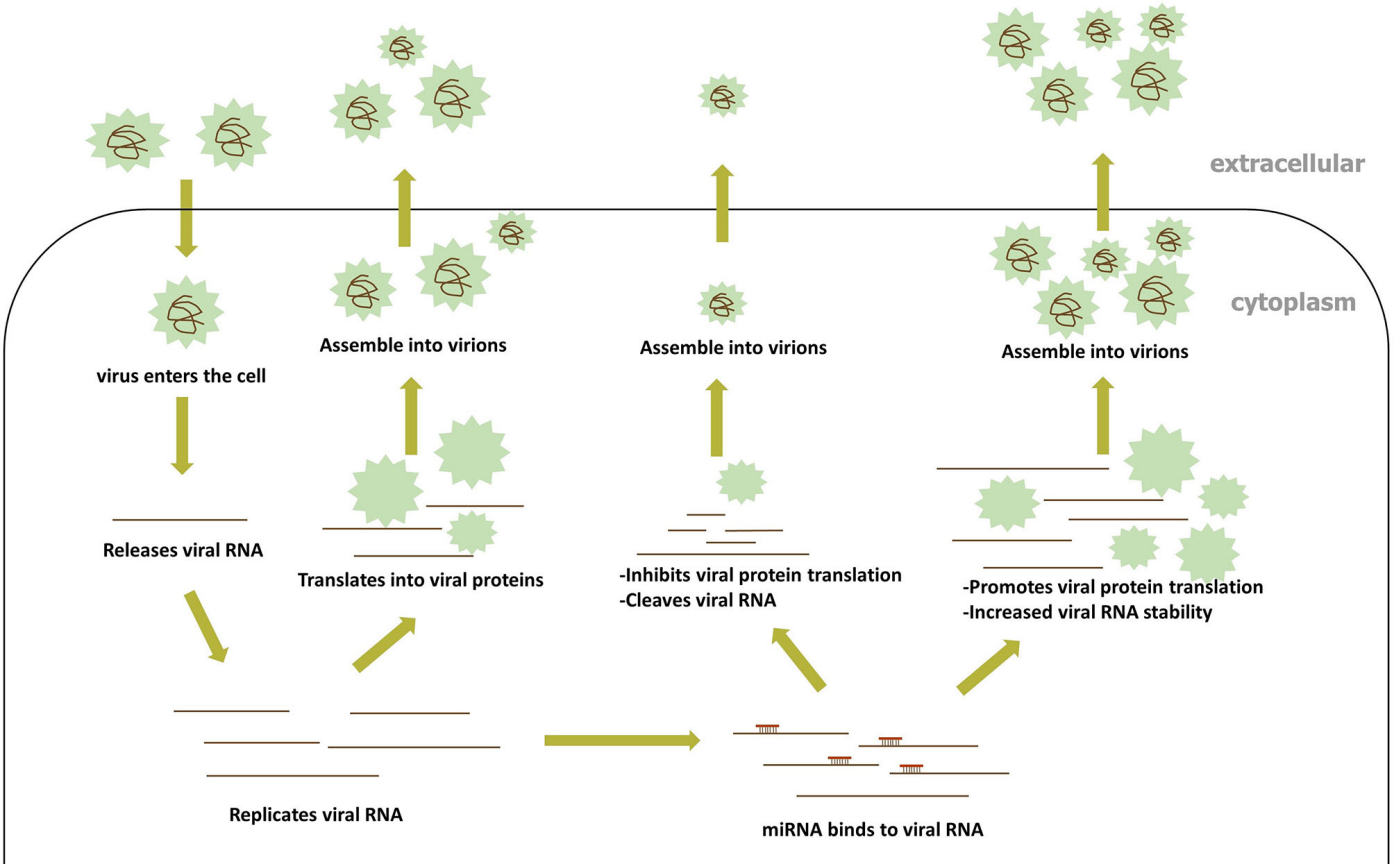
The process of miRNA directly binding to viral RNA.

Accumulating evidence demonstrates that host miRNAs participate in the replication and pathogenesis of the viruses by binding directly to the RNA of many RNA viruses. During RNA virus infection, miRNA can then target RNA virus genes in a manner similar to the way they target host genes (Teterina et al., 2014; Bakre et al., 2017). In general, the 5’UTR and 3’UTR of the RNA virus gene have the most natural binding sites to miRNA, but evidence accumulated toward proving the presence of miRNA-binding sites in the RNA virus protein open reading frame (ORF). A positive-strand RNA virus genome can bind directly to the host’s miRNAs in the same way as host mRNA binding, while the positive-strand intermediate of dsRNA and negative RNA virus work in a similar fashion. The direct targeting of RNA viruses by miRNA encompasses two methods of modulating virus replication (**Figure 3**): (1) the inhibition of viral RNA translation reduces viral replication, and (2) changes in the stability of the viral RNA secondary structure; both methods are discussed in detail below.

### Translation Inhibition by the Host miRNA Binding to the RNA Within the RNA Virus

Many relevant examples have proved that miRNA inhibits viral proliferation and replication by targeting viral RNA and gene silencing. For example, the infectious bursal disease virus (IBDV), a member of the family Birnaviridae, is targeted by gga-miR-21 that downregulates the expression of IBDV VP1 at the translational level rather than the mRNA level, which therefore represses IBDV replication (Wang et al., 2013). Similarly, the virulent newcastle disease virus (NDV) is a negative-strand RNA virus and is translated into viral protein after NDV has been transcribed for a positive-sense RNA upon entry into host cells. gga-miR-1603 and gga-miR-1794 (gga, chicken) were confirmed to target two highly conserved regions of the L gene of NDV, to inhibit the expression of the L protein at both the protein and RNA levels, thus suppressing NDV replication (Chen et al., 2021). Both of these examples are based on the RNA-silencing effects of miRNA in inhibiting protein translation within RNA viruses, and some more interesting examples of miRNA and RNA viruses are mentioned below.

Previously, only the single regulation of host miRNA on the host genes or viral genes was focused on. With further research, miRNA was later found to be able to simultaneously target both the host and virus gene to limit virus proliferation. A few examples to reference this procedure is described below. Studies have shown that gga-miR-130b in DF-1 cells can simultaneously inhibit viral replication by targeting the IBDV segment A and enhance the expression of type interferon by targeting the intracellular cytokine signal suppressor 5 (SOCS5) (Fu et al., 2017). The genomic segment B of IBDV binds to the gga-miR-454 of DF-1 to inhibit virus replication, while cellular cytokine signal suppressor 6 (SOCS6) is inhibited by gga-miR-454 to boost the immune response (Fu et al., 2018). The miR-324-5p directly targets the H5N1 virus PB1 gene and the cellular CUEDC2 gene, the negative regulator of the interferon pathway, to inhibit the gene expression of both genes, thus enhancing innate immunity (Kumar et al., 2018). In these cases, on the one hand, the host miRNA directly blocks the expression of the viral proteins by targeting viral genes, and on the other hand, increases the host immune response by inhibiting the expression of negative immune regulators in the host, in order to concomitantly limit viral proliferation *in vivo*. Another example is the porcine epidemic diarrhea virus (PEDV), which belongs to the genus Alphacoronavirus of the Coronavirus family, a single-stranded, positive-sense RNA virus. It was confirmed that miR-221-5p acted as a dose-dependent inhibitor for PEDV, by directly targeting the 3’UTR of the PEDV genomic RNA, and stimulating NF-κB signaling *via* p65 nuclear translocation, to upregulate IFN-β and related ISGs (Zheng et al., 2018). Due to the mechanism used by miRNAs when binding to the RNA described, and the examples above, miRNAs can simultaneously target both viral and host genes, cocreating an environment that affects viral replication.

In the course of a previously published study, it was found that mutations in miRNA targets within some RNA viruses could render the functions of miRNAs targeting RNA viruses ineffective. These phenomena probably occur because RNA polymerases lack the same collation activity as DNA polymerases and miRNA sequences are highly conserved, thus allowing RNA viruses to mutate and fleetly escape from the host’s miRNA inhibition. For instance, as a member of the Orthomyxoviridae family, the influenza A virus (IAV) is a negative single-stranded RNA virus and has multiple subtype strains. A study verified that hsa-miR- 1307-3p, as a novel potent suppressor, could bind directly to the NS1 RNA of IAV to suppress NS1 expression and influenza virus replication, but a new mutation has been identified in the NS1 gene, which is targeted by hsa-miR- 1307-3p, of more than 100 strain types represented by A(H1N1)pdm09 (a subtype strain of IAV), which invalidated the inhibition of miRNA (Bavagnoli et al., 2019). Analogously, ssc-miR-204 and ssc-miR-4331 only exhibited an inhibitory effect on SIV-H1N1/2009 (a subtype strain of IAV) replication due to mutations in the peer sequences of other strains (Zhang S. et al., 2017). Many similar examples have led to the viewpoint that mutations may represent a means by which RNA viruses evade the direct binding inhibition effects of the host miRNAs. To avoid the influence of gene mutation on the RNA virus protein expression is critical as such mutations are synonymous in many similar findings under natural conditions. Another example, enterovirus 71 (EV71) of the family Picornaviridae, a single-strand positive-sense RNA virus, could have its VP1 and VP3 protein levels regulated by hsa-miR-296-5p to inhibit virus infection (Zheng et al., 2013). Whereas, by aligning the sequences of hsa-miR-296-5p binding to the genome in each EV71 subtype, some strains were found to have synonymous mutations in the same location, research confirmed that these viruses could escape the suppression of hsa-miR-296-5p (Zheng et al., 2013). However, many RNA viruses are still inhibited by the host miRNA. The randomness and uncertainty of mutations may make correct mutations less efficient. Moreover, the short-term inhibition of miRNAs on virus replication may not hurt or hinder the entire process of virus proliferation, so not all RNA viruses need to escape the effects of miRNAs. Naturally, miRNA-binding sites in RNA viruses that create no positive effects on viral replication are more likely to have been deleted *in vivo* than miRNA-binding sites that have positive effects on viral replication.

Given the evidence pointing toward the importance of binding sites, scholars studied the highly conserved miRNA-binding sites in RNA viruses. It was found that the antiviral activities of miRNAs caused by direct targeting RNA viruses may be used by the virus to evade immunity, prolong incubation periods, or even increase the virulence of the virus during evolution. Under some conditions, the downregulation of virus replication induced by miRNA may in fact be necessary for the persistence of viral infection (Mahajan et al., 2009). Studies have shown that the eastern equine encephalitis virus (EEEV) utilized the inhibitory effect of miRNAs to prevent the virus from being detected by the immune system prematurely, resulting in the enhanced neurovirulence of the virus. EEEV, a single-stranded positive-sense RNA mosquito-borne alphavirus, encodes four strongly conserved miRNA-binding sites for has-miR-142-3p in myeloid-lineage cells (Trobaugh et al., 2014). The hematopoietic cell-specific miR-142-3p restricts EEEV replication to determine virus tropism and to suppress innate immune responses in the myeloid cell, which is conducive to microbial infection and exacerbates EEEV neurovirulence (Trobaugh et al., 2014). The presence of the miRNA-binding sequences is necessary for efficient EEEV replication in mosquitoes, so it is legitimate to assume that these sites are essential for virus transmission (Trobaugh et al., 2019a). For EEEV, the number of miR-142-3p-binding sites is dominant in the suppression of EEEV replication. Even though the miRNA inhibits EEEV expression, the inhibitory effect of miRNA on EEEV is very important for both prolonging the incubation period and increasing virulence during the whole virus proliferation process *in vivo*. Besides this, after human immunodeficiency virus type 1 (HIV-1)- infected resting primary CD4+ T cells, the significantly enriched miRNAs including, miR-28, miR-150, miR-223, and miR-382 potently inhibited HIV-1 production by targeting the 3’UTR of the HIV-1 RNA and related host proteins (Huang et al., 2007). The inhibition of related host proteins including Tat and Rev, which are key factors for transcription and translation in viral RNA, could further strengthen the viral incubation period to help the virus multiply (Huang et al., 2007). Additionally, studies have shown that miR-326, miR-196b, and miR-1290 have similar effects (Houzet et al., 2012; Wang et al., 2015). The chikungunya virus (CHIKV) is a positive-sense single-stranded RNA virus in the *Alphavirus* genus, transmitted by *Aedes aegypti* (*Ae. aegypti*) mosquitoes. *Ae. aegypti* miR-2944b-5p can bind to the 3’UTR of CHIKV and reduce CHIKV replication, meanwhile regulating the cellular target, vps-13 (vacuolar protein sorting) (Dubey et al., 2019). CHIKV may be using miR-2944b-5p, alongside its target vps-13, to maintain the cellular integrity of the mitochondrial membrane potential, therefore helping the CHIKV survive in mosquito cells (Dubey et al., 2019). This miRNA can inhibit excessive proliferation of CHIKV in the intermediate host, and maintain the titer of the virus in the intermediate host together with the genes of the intermediate host *in vivo*, which also contributes toward the transmission of the virus. In general, the highly conserved miRNA-binding sites in RNA viruses have positive implications for virus survival.

### miRNA Influence Upon the Spatial Structure of Viral RNA

It is already known that RNA has a certain spatial structure under natural conditions. This did lead to the question as to whether the binding of host miRNA to viral RNA could alter viral RNA to enable interactions with other proteins by changing the spatial structure of viral RNA once the RNA virus enters the host cell to begin replication or translation. There have been some specific examples that have helped elucidate this matter. The hepatitis C virus (HCV), a single-stranded RNA virus with the Flaviviridae family, only has an ORF and requires liver-specific miR-122 interactions with the sequences from the HCV RNA 5’UTR to maintain a high viral RNA abundance in the liver (Jopling, 2008). The 5’UTR and 3’UTR of the HCV RNA have four and three SL structures, respectively, and the ORF region encodes four structural proteins and six non-structural proteins (Jopling, 2008). Two miR-122-binding sites in the 5’UTR, which are separated by a highly conserved 14-nucleotide sequence, are occupied by miR-122 to synergistically give HCV RNA higher stability rather than inhibiting viral replication through RNAi-related functions (Jopling, 2008). Some studies have suggested that efficient HCV replication require the annealing binding of miR-122 to these two sites, which then forms a trimolecular RNA structure that is essential for efficient virus proliferation (Amador-Cañizares et al., 2018; Chahal et al., 2019). However, some scholars believe that any small RNA binding to the miR-122-binding sites on the HCV 5’UTR can promote the HCV life cycle (Kunden et al., 2020). Furthermore, some researchers have found that the insertion of the viral miR-122-binding site into the 3’NCR of a reporter mRNA leads to the downregulation of mRNA expression, which implies that the location of the miRNA-binding site may dictate its effects on gene regulation (Jopling, 2008; Jopling et al., 2008). Further research has shown that miR-122 binds to the 5’NTR of the HCV gene, to protect uncapped HCV RNA genes by defending the degradation caused by 5’ exonuclease Xrn1 and Xrn2, whereas miR-122 negatively regulates the expression of the reporter mRNA by binding to the 3’NCR of the mRNA in a general manner (Li et al., 2013; Sedano and Sarnow, 2014). This interaction promotes HCV RNA accumulation by stabilizing viral RNA, leading to changes in the secondary structure of the viral genome, stimulating the generation of the canonical HCV IRES RNA structure (Schult et al., 2018; Chahal et al., 2019; Kunden et al., 2020). The multiple effects of miR-122 on HCV proliferation are based on the changes observed to the HCV secondary structure after miRNA has bound to viral RNA. Some similar examples are also described in other systems. When the dengue virus (DENV) infects human cells *in vitro*, endogenous miR-548g-3p can bind to the conserved stem-loop promoter (SLA) (a promoter for DENV RNA transcription) in the 5’NTR of the DENV genome to suppress DENV intracellular replication (Wen et al., 2015). Based on this study, it can be inferred that the combination of miR-548g-3p and the targeted site of DENV, the natural SL structure of the promoter element needed to regulate the binding efficiency of DENV promoter and RNA-dependent viral RNA polymerase may be destroyed (Yu et al., 2008; Wen et al., 2015). Besides this example, the bovine viral diarrhea virus is also targeted by miR-17 and let-7 through binding to S2 and S1 present in the virus genome 3’-NTR, respectively (Scheel et al., 2016; Kokkonos et al., 2020). AGO2 and miR-17 binding were essential for viral replication, whereas let-7 binding increased virus translation.

These examples indicate the importance of the miRNA binding site location for targeting the virus RNA. If the binding site is located in the folding of the secondary structure of RNA, SL structure, or the binding site of some proteins, the corresponding miRNA may have an important influence on the expression or function of this genome (Gerresheim et al., 2017). Interestingly, the unconventional relationship between miRNA and viral RNA has also been found in some other RNA viruses, which most or all enhancing the stability of the viral RNA to positively regulate RNA virus replication.

## Conclusions

Eukaryotic miRNAs participate in the regulation of most physiological activities within cells, including RNA virus infection. RNA viruses use RNA as a gene carrier, and miRNAs bind to RNA in a base complementary way, which allows some miRNAs to directly target the RNA within RNA viruses. Most of this binding occurs in the ORF or 3’UTR of the RNA virus RNA, whereas miRNA works with RNA-binding proteins to inhibit RNA viral replication. In addition, some cellular miRNAs are able to bind to RNA viral RNA to avoid the cleavage of the related enzymes or to stabilize the spatial structure of the RNA to positively regulate RNA virus replication. However, as there is a lack of any proofreading function, RNA viruses can escape from miRNA through gene mutation in the face of the miRNA’s direct targeted inhibition. From the above-cited publications, which include many examples, on the one hand, RNA viruses utilize some specific cellular miRNAs to create an intracellular environment conducive to the transmission of viral infection. Yet, on the other hand, it is commonplace that most miRNAs that directly or indirectly strongly inhibit RNA virus replication are manipulated by the RNA virus to reduce expression, or are shunned by RNA viruses with genetic mutations. Moreover, miRNAs in eukaryotes are highly conserved and are not easily mutated, which makes it difficult for miRNAs to be dominant antiviral factors against RNA viruses in nature.

Research has shown that it is feasible for researchers to insert the binding sequences of intracellular specific miRNA into the RNA virus genes in order to prepare miRNA vaccines according to the inhibition of targeted RNAs by some miRNAs (Tsetsarkin et al., 2015). The insertion of single or multiple copies of the brain-expressed miRNA target sequence in the 3’NTR of the genome of the neurotropic chimeric tick-borne encephalitis virus/dengue virus 4 (TBEV/DEN4) flavivirus could reduce the neurotoxicity of the virus (Heiss et al., 2012). However, the artificial RNA virus can also escape from miRNA to regain its neurotoxicity by accumulating gene mutations or *via* deletion (Heiss et al., 2012). This phenomenon can be alleviated by increasing the copy number of inserted sequences and setting appropriate spacing. Similarly, the deletion of the miR-142-3p-binding sites in the EEEV 3’ UTR can lead to efficient EEEV infection of myeloid cells, resulting in a decrease in the virulence of the virus, but the accumulation of genetic mutations may restore virus virulence during serial passages of the virus (Trobaugh et al., 2019b). How to stabilize the artificial sequence while also inhibiting the virulence of the virus needs further research. Host miRNA is hijacked by invading RNA viruses and participates in the pathogenic process of viruses, or miRNAs inhibit RNA virus replication by targeting their RNA, which makes it reasonable for miRNA to be a potential new therapeutic target for RNA viral therapy. There have been attempts to develop antiviral drugs based on miRNA studies. In the laboratory, artificial miRNAs are designed to target the conserved genomic regions of the West Nile virus NS5 and NS2A or consensus sequence of 3’NTR of JEV, and all show effective suppression of the virus *in vitro* (Sharma et al., 2018; Karothia et al., 2020). Scientists have tried to use similar RNAi technology to treat some viral diseases in clinical trials, such as the inhibition of HBV replication in chronic HBV infection (Wooddell et al., 2017; van den Berg et al., 2020), treating Ebola virus patients to improve survival and recovery rates (Thi et al., 2015) and targeting HIV-1 replication for cell transplant therapy (Scarborough and Gatignol, 2017). It is not only miRNAs in animals that can limit the influence of RNA viruses, as miRNAs in plants have also been found to inhibit the replication of certain viruses. MiR-2911 from honeysuckle (a traditional Chinese medicine) can suppress IAVs with a broad spectrum by directly targeting the IAV RNA, and the let-7a from this plant can inhibit the replication of DENV by targeting its NS1 sequence (Zhou et al., 2015; Lee et al., 2017).

Intracellular miRNA, as a link of the intracellular regulatory network, can simultaneously target multiple cellular mRNAs and the RNA of the invading RNA virus. Cellular miRNAs, the host mRNA, and RNA viruses are closely related and interact with each other, affecting the whole body. This connection makes miRNAs a potential breakthrough in the future to tackle the current ravages of many RNA viruses. However, in order to be an antiviral drug, miRNA requires comprehensive consideration for the variable of cell homeostasis and other physiological conditions caused by changes in the miRNA. Moreover, a large number of convenient, cheap, and accurate synthesis methods of artificial miRNA still need further study. At present, miRNA as a new approach against RNA viruses is still far from being realized, and a breakthrough in the field is expected.

## Author Contributions

LL was responsible for the collection and analysis of materials and organizing and writing the manuscript. RJ, AC, and MW gave the main modification reviews and suggestions. All authors contributed to the article and approved the submitted version.

## Funding

This work was supported by the National Natural Science Foundation of China (31872475), Sichuan Veterinary Medicine and Drug Innovation Group of China Agricultural Research System (CARS-SVDIP), and China Agricultural Research System (CARS-42-17).

## Conflict of Interest

The authors declare that the research was conducted in the absence of any commercial or financial relationships that could be construed as a potential conflict of interest.

## Publisher’s Note

All claims expressed in this article are solely those of the authors and do not necessarily represent those of their affiliated organizations, or those of the publisher, the editors and the reviewers. Any product that may be evaluated in this article, or claim that may be made by its manufacturer, is not guaranteed or endorsed by the publisher.
